# Indicators of Safety and Wellbeing in Patients Starting Maintenance Haemodialysis Using Phased Approach: Findings from a Cohort Feasibility Study

**DOI:** 10.3390/healthcare14091117

**Published:** 2026-04-22

**Authors:** Adil M. Hazara, Maureen Twiddy, Victoria Allgar, Sunil Bhandari

**Affiliations:** 1 Department of Renal Medicine, York Hospital, York YO31 8HE, UK; 2 Hull York Medical School, University of York, York YO10 5DD, UK; sunil.bhandari@nhs.net; 3 Institute of Clinical and Applied Health Research, University of Hull, Hull HU6 7RX, UK; maureen.twiddy@hyms.ac.uk; 4 Peninsula Clinical Trials Unit, Faculty of Health, University of Plymouth, Plymouth Science Park, Plymouth PL6 8BX, UK; victoria.allgar@plymouth.ac.uk; 5 Academic Renal Research, Hull University Teaching Hospitals NHS Trust, Hull HU3 2JZ, UK

**Keywords:** phased haemodialysis start, kidney failure, dialysis treatment, feasibility study, incremental HD

## Abstract

**Background:** The optimal method of starting maintenance haemodialysis (HD) in patients with kidney failure is not known. We have compared early treatment characteristics, blood pressure trajectories, and selected dialysis-related safety events in patients who started HD using a stepped and phased approach, with those who received conventional care. **Method:** A single-centre cohort feasibility study was conducted. Participants with kidney failure, about to start maintenance HD, were enrolled prospectively (intervention arm). They started treatment on a novel regime comprising four pre-specified incremental steps (Phases 1 to 4) over 14 weeks. They were matched using propensity scores with historical controls: patients who had previously started HD on a three-times weekly basis from the outset (control arm). **Results:** The final cohort comprised 15 and 29 participants in the intervention and control arms respectively (1:2 ratio; one control excluded after matching). Intervention group participants were slightly older with a higher proportion of men. The rate of decline in blood pressure was slower in the intervention group. There were also signals for fewer events of intra-dialytic hypotension (211 vs. 379 per 100 person-year), infections not requiring admission (56 vs. 114 per 100 person-year) and loss of vascular access (56 vs. 79 per 100 person-year) in intervention group. There was a signal for higher incidence of severe hypertension (systolic BP ≥ 180 or diastolic BP ≥ 110 mmHg) in the intervention group. Hospitalisation rates were similar; there were no deaths and one non-fatal major cardiac event (MACE) in the intervention group, and one death and no MACE in the control group. **Conclusions:** Implementing a short transitional regime of incremental HD may be possible in clinical settings, potentially helping to reduce the gradient of physiological change and burden of early treatment. The findings of this feasibility study are exploratory, and fully powered randomised controlled trials are needed to establish the efficacy and safety of such a programme.

## 1. Introduction

In patients with advanced chronic kidney disease (CKD), the optimal strategy for starting long-term in-centre haemodialysis (HD) therapy remains unclear. The convention of starting treatments at three times (3×) weekly sessions from the outset has long been accepted as the standard of care [1,2]. This approach, however, has faced mounting calls for revision [3,4] as more evidence comes to light, of the difficulties patients face soon after starting HD therapy. Mortality rates are high in patients transitioning into long-term dialysis [5,6], whilst the start of conventional treatment is associated with worsening of functional status and a decline in quality of life.

To improve early outcomes in patients making the transition to long-term HD, a more stepped and phased initiation of treatment may be preferrable. Most patients who start treatment still have residual kidney function (RKF) and may remain well on less than 3× weekly treatments [7]. The reduction in treatment burden could offer patients with CKD a less physiologically challenging route to their establishment on long-term treatments as well as help improve early outcomes. Starting HD at a reduced frequency has been shown to be associated with better preservation of RKF compared to standard care [8].

Various strategies have been proposed on how to implement HD with reduced frequency in clinical practice [9]. The term ‘incremental HD’ has been used to mean dialysis with reduced frequency (usually twice [2×] weekly), with regular monitoring of RKF and planned increments in treatment times in response to declining residual function. The requirement for urine collections for RKF measurements are important in these regimes and ensures adequacy of HD. Urine output measurements can, however, be difficult, and adds a further layer of complexity; certain patient groups, for instance more frail individuals and those with cognitive impairment, may not be able to reliably provide timed urine collections [3]. Including them in incremental HD programmes is critical in improving early outcomes.

We have previously published the design of a short transitional regime of incremental HD [10] aimed at supporting patients in the initiation of long-term therapy. In developing this regime, we hypothesised that an incremental HD programme may be delivered in a time-limited manner without relying on urine collections, instead using conventional measures for safety monitoring. We conducted a mixed-method study with the primary objective of testing the feasibility of recruitment and retention of participants into our programme [11]. Our aim, ultimately, was to pave the way towards addressing the existing knowledge gap regarding the safety and clinical impact of short and pragmatic transitional haemodialysis start programmes.

In the current paper, we report the secondary outcomes from our feasibility study. These outcomes were all exploratory in nature. Our objectives were to compare key indicators of patient wellbeing in our short transitional regime of HD vs. conventional care. These indicators included early blood pressure control, intra-dialytic fluid gains, biochemical profile, hospitalisations, cardiovascular events, and mortality during a six-month follow-up period.

## 2. Materials and Methods

### 2.1. Study Design and Participants

We conducted a single-centre cohort study with a prospectively recruited intervention arm who started dialysis on a twice-weekly basis with pre-planned increases in treatment duration and frequency (see below). Participants in the intervention arm were matched with historical controls (matching ratio 1:2) from a register of patients who previously started HD in the conventional way. Full methods for the current study have previously been published [10]. Adults with CKD stage 5 (from any cause) commencing in-centre maintenance HD therapy for established kidney failure in the out-patient settings were eligible (see eligibility and exclusion criteria in Supplementary Table S1). Participants were followed up for six-months after the start of HD.

### 2.2. Recruitment

Recruitment in the intervention arm was completed over two periods interrupted by the first wave of the COVID-19 pandemic. Participants were enrolled from either the pre-dialysis specialist clinics or the out-patient dialysis unit. Following enrolment, participants remained under follow-up at their usual clinics until the point of starting HD. The timing of HD initiation was made independently of the study investigator by the clinical care teams, based on clinical needs and in consultation with patients and their relatives. Participants for the control arm were identified using the local dialysis treatment database Euclid (Fresenius Medical Care, Bad Homburg, Germany). All patients starting HD therapy since 2013 (the inception date of the database), who met the above eligibility criteria, were included in the matching run.

### 2.3. Matching and Sample Size

Propensity scores matching (PSM) was undertaken to match participants in the treatment group with two historical controls. PSM was performed using 13 variables: age, serum albumin, vascular access type, pre-dialysis serum creatinine, dialysis facility, body mass index (BMI), parathyroid hormone levels (PTH), diastolic blood pressure, haemoglobin, systolic blood pressure, vitamin K antagonist treatment, history of cardiovascular disease, and antihypertensive treatment [12]. These variables, along with C-reactive protein (CRP), together predicted the 2-year mortality in dialysis patients with sensitivity and specificity of 72% and 69%, respectively [12]. CRP levels are not routinely performed in new HD starters at our institution and were not matched. We used optimal matching of propensity scores to minimise within-pair differences and applied the nearest neighbour method (no calliper range specified) with no replacements [13]. We did not specify calliper range as the number of participants in the invention group was low; imposing a calliper would have reduced the number of matched pairs. We have presented standard mean difference to assess the adequacy of matching.

As this was a feasibility study, a formal sample size calculation was not performed. An initial target of 80 participants (40 participants in each arm) was set for pragmatic reasons based on the expected number of new dialysis starters at our institution [14]. The study was severely impacted by the first wave of the COVID-19 pandemic and had to be temporarily stopped due to staff relocation to cover vital services. When the study recommenced, restrictions were still in place in terms of patient contact, and the recruitment target was revised down to 20 participants in the intervention arm, though we kept the same number of controls and adopted a 1:2 matching. Both the original and revised protocols were approved by the research ethics committee (the West of Scotland Research Ethics Committee-4, Ref: 19/WS/0019).

### 2.4. The Intervention

Participants in the intervention arm started therapy on a 2× weekly basis with progressive increases in the duration and frequency of sessions over 14 weeks and 2 days, achieving conventional treatment times by the end of this period. The intervention has been described in detail previously and is presented further using the Template for Intervention Description and Replication (TIDieR) [15] (see Supplementary Table S2).

### 2.5. Follow-Up and Data Collection

All participants were followed up for six months from the start date of HD therapy or until death, change in kidney replacement therapy modality, or change of centre. Dialysis treatment data (including nursing notes and exceptions), along with details of medical history were extracted. Routinely, all dialysis treatments are recorded in the dialysis treatment database Euclid (Fresenius Medical Care, Germany). At the end of each dialysis session for any given patient, a named nurse documents a narrative account of that day’s dialysis treatment and makes notes of any significant events. This information was accessed and interrogated for pre-defined safety events (see Table 1) and substantiated with information documented elsewhere in patients’ electronic health records. Weight and blood pressure readings were monitored at baseline and then at each subsequent dialysis visit. BMI was calculated using patients’ post-dialysis weights. Urea reduction ration (URR) and dialysis eKt/V_urea_ were calculated monthly using the pre- and post- dialysis serum urea levels. As previously reported [11], urine collections were performed in the intervention group at baseline and at 1, 3, and 6 months. These data were used to calculate the renal contribution to weekly std-Kt/V using the Solute Solver equation [16].

### 2.6. Outcomes and Analysis

Baseline characteristics of participants are summarised as means with standard deviations (SD) or proportions as appropriate. Characteristics of dialysis treatment, namely: session length, frequency, and Kt/V readings from each session, are summarised using descriptive statistics. For the analyses/comparisons of measurements taken at each session, the follow-up interval was split according to the phases of incremental HD regime; hence a phase-wise comparison is presented for these measurements. The dataset of observations from dialysis sessions had a highly hierarchical and unbalanced structure. Multi-level mixed linear regression was carried out to produce estimates of means and 95% confidence intervals (CI) for such measurements (e.g., for pre-dialysis blood pressure readings). Estimates have been adjusted for age and gender. This is because, despite PSM, there were residual differences in these key demographic features. For analyses of measurements taken monthly, month-wise comparisons are presented between the intervention and control groups. Although less hierarchical than the sessional data, these observations also resulted in an unbalanced dataset. As above, multi-level mixed linear regression analyses were carried out to determine population means and 95% CI; estimates were adjusted for age and gender.

Incidence rates were calculated by dividing the number of events by the person-time. Incidence rate ratio was then calculated with 95% CI for the point estimate. In the incidence rate calculations, we used events as the unit of analyses to capture the overall burden of events. This approach allowed inclusion of recurrent events experienced by the same participant, which would have been underestimated if analyses were restricted to the first event per participant. The number of participants experiencing each event is also reported in a table to provide additional context.

Numbers of deaths from all causes, hospitalisations and the 4-point major adverse cardiovascular events (4p-MACE: a composite of cardiovascular deaths, nonfatal myocardial infarctions, nonfatal strokes, and hospitalisations for unstable angina) have been reported for both the treatment arms. Due to the low numbers of deaths and MACE, they are described in the narrative section of the results but not formally compared.

*p* < 0.05 was considered significant for all analyses. Data were collected and organised using MS Excel (Microsoft Corporation, Redmond, WA, USA) and statistical analyses were completed using Stata BE 17 (Statacorp LLC., College Station, TX, USA).

## 3. Results

### 3.1. Participant Characteristics

Analyses included 44 participants: 15 in the prospective intervention arm and 29 matched historical controls (originally 30, one patient was excluded after matching records showed that they were an inpatient HD starter and hence would not have met the inclusion criteria). Baseline characteristics of the intervention and control group patients are presented in Table 2. The timeline for enrolling participants in the intervention arm is presented in Supplementary Figure S1.

Participants in the intervention group were slightly older compared to the control’s and contained a higher proportion of males. They also had a longer history of specialist input prior to commencing dialysis. Other laboratory and clinical parameters were well matched.

### 3.2. Treatment Characteristics: Duration and Adequacy

**Figure 1:**
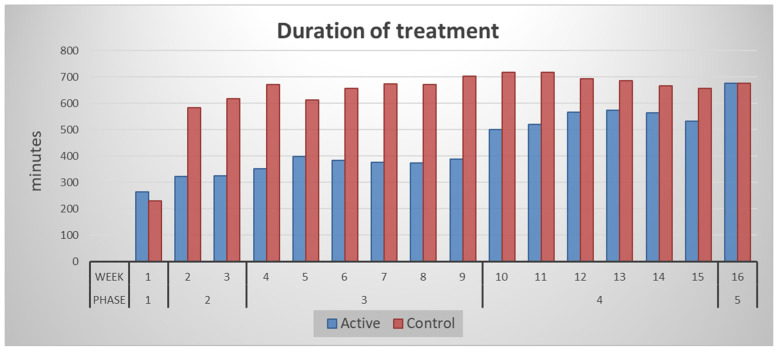
Duration of dialysis therapy in the incremental (active) and control groups, presented per week and per phase of the study. Total treatment times increased as the study progressed in the incremental group, whilst they remained steady in the control group.

Treatment times and weekly standard Kt/V-urea progressively increased in the intervention group as planned (Figure 1).

**Figure 2:**
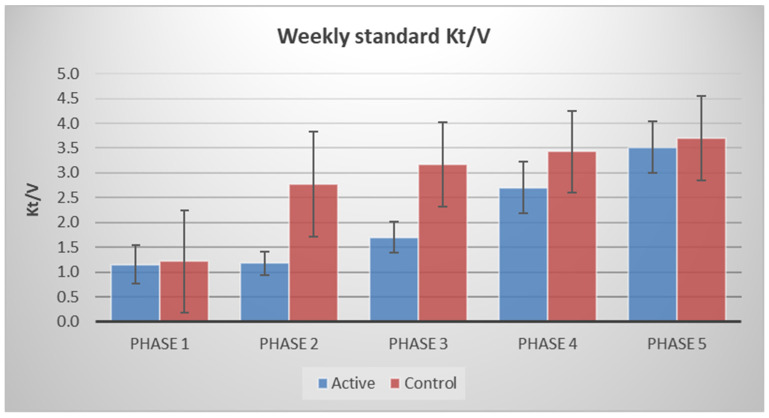
Mean standard weekly Kt/V-urea in each phase in the incremental (active) and control groups. Note that these figures do not account for contribution of residual renal function.

In the period spanning over all four phases of the short incremental regime, participants in the intervention group received a mean (SD) of 6249 (717) hours of dialysis, 30% lower than the 8898 (2622) hours administered to the control group over the same period. Weekly standard Kt/V-urea was 56%, 46%, and 21% lower during Phases 2, 3 and 4, respectively, in the intervention group (Figure 2).

Over the four phases of treatment, the total time spent receiving HD treatment was 30% lower in the intervention group compared to matched controls. In the intervention group, when the contribution of renal-Kt/V was accounted for, the mean weekly std-Kt/V was above 2.1 during all phases of the incremental treatment. Mean weekly standard Kt/V in this group, achieved through a combination of dialysis-Kt/V and renal-Kt/V, was 2.2 (95% CI 1.9–2.4) at baseline, rising to 2.5 (95% CI 2.2–2.8), 2.7 (95% CI 2.4–3.0), and 3.0 (95% CI 2.7–3.3) at months 1, 3, and 6, respectively, since the start of dialysis.

### 3.3. Blood Pressure Control

**Figure 3:**
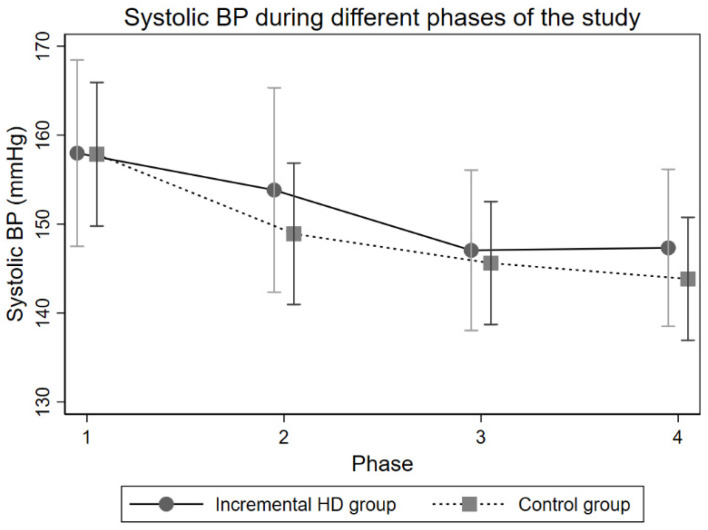
Mean systolic blood pressures in the incremental HD and control groups. Adjusted for age and gender. Error bars represent 95% confidence intervals.

Participants in the intervention group had similar blood pressures at the outset compared to controls; mean (SD) systolic blood pressures 158/74 (28/9) mmHg vs. 157/72 (28/16) mmHg, respectively. As the treatment continued, blood pressures decreased in both groups (Figure 3).

Mean drop in systolic blood pressure from baseline in the incremental HD group was −4 (95% CI −16–8) mmHg, −11 (95% CI −21–−1) mmHg, and −11 (95% CI −20–−1) mmHg during Phases 2, 3 and 4, respectively. For the same periods, the mean drop in BP from baseline in the control group was −9 (95% CI −17–−1) mmHg, −12 (95% CI −20–−5) mmHg, and −14 (95% CI −21–−7) mmHg, respectively. Hence, there was a signal for steeper decline in systolic blood pressure in the conventional treatment group, particularly during the early phases (Phases 1 and 2) of the incremental treatment.

Note that the data presented in this section reflect pre-dialysis blood pressures taken after the ‘long gap’ in the weekly dialysis schedule in both groups. This approach enabled us to compare same number of readings per participant.

### 3.4. Safety

**Figure 4:**
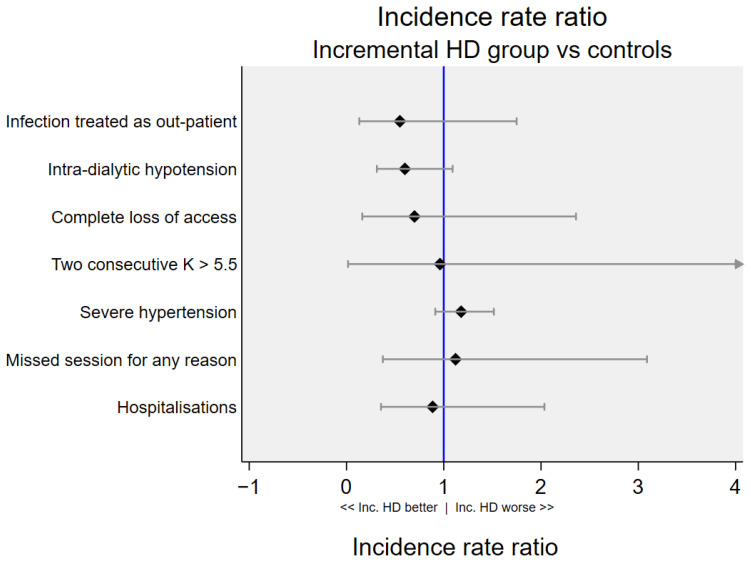
Incidence rate ratio of adverse events in the incremental HD group compared to controls. Error bars represent 95% confidence intervals; inc. HD: incremental haemodialysis. Note that this feasibility study was not powered to show significant differences in incidence rate between the two treatment groups.

Adverse events recorded for participants in the two treatment arms are summarised in Table 3, and the incidence rate ratio for these events are presented in Figure 4.

#### 3.4.1. Infections Treated as Out-Patient

There were 4 infections (treated without need for hospitalisation) affecting three participants in the intervention group and 14 infections affecting nine participants in the control group. The incidence rate was lower in the intervention group compared to the controls (56.3 vs. 102.4 per 100 person-year, respectively); incidence rate ratio 0.5 (95% CI 0.1–1.7). In the intervention group, indications for antibiotic treatment were urine (three events) and line/exit site infections (one event). In the control group, treatments were given out for infections at lower leg 4, line/exit site 4, urine 2, chest 1, unspecified wound 1, finger 1, and unknown 1 (infections leading to hospitalisations are presented below).

#### 3.4.2. Intra-Dialytic Hypotension

There were 15 episodes of intra-dialytic hypotension affecting seven participants (47% of participants) in the intervention group (incidence: 211.0 per 100 person-year) and 48 episodes affecting 18 participants (62% of participants) in the control group (incidence: 351.1 per 100 person-year). The incidence rate ratio for intra-dialytic hypotension was 0.6 (95% 0.3–1.1) in favour of the intervention group.

#### 3.4.3. Dialysis Access

Complete loss of access, including fistula loss and change of central venous catheters (CVC) due to dysfunction, occurred in four instances involving three participants (20% of all participants; incidence 56.3 per 100 person-year) in the intervention group and in 11 instances involving nine participants (31% of all participants; incidence 78.6 per 100 person-year) in the control group; incidence rate ratio 0.7 (95% CI 0.2–2.4). There were four instances in each group (one event per person in each group) when the fistula required resting without the need for new access, mostly for extravasation of blood (causing localised haematoma). The incidence was higher in the intervention group (56.3 vs. 29.3 per 100 person-year), with an incidence rate ratio of 1.9 (95% CI 0.4–10.3) in favour of conventional treatment.

#### 3.4.4. Hyperkalaemia

Due to the study design, there were more potassium readings available for the intervention group (193 readings for 15 participants; 12.9 readings per participant) compared to the control group (266 readings for 29 participants; 8.9 readings per participant). Hence, the potential for detection bias in favour of conventional treatment, due to fewer available readings, cannot be excluded.

Potassium control was acceptable in both groups and there were no signals for harm.

At month 1, there was signal for a slightly higher serum potassium level in the intervention group—mean potassium 4.5 (95% CI 4.2–4.8) mmol/L vs. 4.3 (95% CI 4.0–4.5) mmol/L in the intervention and control groups, respectively. As treatment progressed, potassium levels were more closely matched.

There were no episodes of severe per-dialysis hyperkalaemia (K > 6.5 mmol/L) in either group. Persistent hyperkalaemia was defined as two or more consecutive readings of >5.5 mmol/L at any point during follow-up. There was one such episode in the intervention group and two in the control group—incidence rate of 14.1 vs. 14.6 per 100 person-year, respectively; incidence rate ratio: 1.0 (95% CI 0.0–18.5).

#### 3.4.5. Severe Pre-Dialysis Hypertension

Incidence of severe pre-dialysis hypertension, defined as systolic blood pressure > 180 mmHg or diastolic blood pressure > 110 mmHg was higher in the intervention group compared to the control group (1448.6 vs. 1229.0 per 100 person-year), although this was not statistically significant; incidence ratio: 1.2 (95% CI 0.9–1.5).

Note that, in contrast to the analyses presented previously (Section 3.3), where only the readings taken pre-dialysis after the longest gap in weekly dialysis schedule were included, the analyses presented in this section included all available pre-dialysis blood pressure readings; 992 sessions in the intervention group and 2044 sessions in the control group.

#### 3.4.6. Hospitalisations, Major Cardiovascular Events, and Deaths

In total, there were nine episodes of emergency hospitalisation involving five (33%) of the 15 participants in the intervention group (incidence: 126.6 per 100 person-year) and 19 episodes involving 14 (48%) of the 29 participants in the control group (incidence: 139.0 per 100 person-year). Hence, the incidence rate ratio for hospitalisation was 0.9 (95% CI 0.4–2.0).

Reasons for hospitalisations in each group are summarised in Supplementary Table S2. Infections were the most common reason for hospitalisation, accounting for 14/29 (48%) of all admissions, followed by requirement for urgent sort-out of dialysis access (5/29 [17%]), chest pain (3/29 [10%]), breathlessness (3/29 [10%]), and other causes (4/29 [14%]). There was one COVID-19-related hospitalisation in the intervention group. The control group was notable for higher numbers of unplanned hospitalisation for urgent dialysis access sort-out; five admissions in the control group vs. 0 in the intervention group.

There was one non-ST elevation myocardial infarction (NSTEMI) recorded in the intervention group and none in the control group. The participant involved sustained the NSTEMI at 4.7 months (20 weeks) after the start of dialysis treatment. There were no deaths in the intervention group and one death in the control group during the first six months of dialysis treatment. The only death in the control group occurred at 3.4 months (14 weeks) after the start of dialysis treatment from sepsis.

## 4. Discussion

We have presented findings from a feasibility study comparing outcomes in people starting HD in a stepped and phased manner, with those starting 3× weekly HD from the outset. There were signals that this short incremental programme was superior to conventional care for intra-dialytic hypotension, infections as treated outpatient, and with loss of vascular access. The intervention was associated with a slower rate of decline in BP over the first few weeks of dialysis; however, episodes of severe hypertension (systolic BP > 180 or diastolic BP > 110) were also more frequent. Hospitalisation rates were similar in the intervention and conventional treatment groups, whilst deaths and non-fatal cardiovascular events could not be compared due to low number of events.

This study was not powered to show significant differences in the pre-specified safety events between incremental HD and control groups; the comparisons presented here are for exploratory purposes, and to detect safety signals. Of the outcomes reported under safety events, a greater degree of confidence may be placed on findings related to intra-dialytic hypotension, severe hypertension (systolic BP > 180 mm Hg), and hospitalisations; this is because the number of events encountered for these were relatively high.

BP dropped significantly in both groups during the follow-up period as expected [17,18]. However, there was signal for a slower drop in BP in the intervention group. Sipahioglu et al. [18] examined blood pressure slopes (rate of changes in blood pressure) in the first 12 weeks of dialysis; those with the greatest rate of decline in blood pressure had a significantly higher mortality risk at year one. The observation of a slower decline in blood pressure supports the rationale for a phased introduction of HD treatments as a way of reducing the gradient of physiological change. There were fewer episodes of intra-dialytic hypotension associated with this strategy, which is also a positive signal in this regard.

Severe pre-dialysis hypertension was common in both groups, affecting 12 of 15 (80%) and 20 of 29 (69%) participants in the treatment and control groups respectively; there was a signal for a higher incidence rate ratio in the treatment group (see Figure 4). This observation complements the above findings of slower rate of decline in BP in the treatment group. It can be postulated that this is related to overhydration and salt load in the less frequent dialysis group. The clinical significance of this is not known; furthermore, due to the short follow-up period we cannot speculate on the impact of phased introduction of dialysis on long-term BP control. In a future RCT, ambulatory interdialytic BP or home BP measurements may be possible in a subset of participants to enable a more accurate assessment of BP control [19] in both groups. The absolute number of events for ‘fistula requiring resting’ was higher in the intervention group. The decision to rest fistula usually follows an event, such as extravasation of blood from the needling site. In ‘resting’ the fistula and deferring dialysis, clinicians routinely account for the urgency of the dialysis treatment based on safety of biochemical parameters and hydration status; more resting could suggest that clinical staff felt it was safe to defer dialysis.

There were no signals for differences in hospitalisation rates between the intervention and conventional treatment groups; this contrasts with recent findings of 69% reduction in hospitalisation rates in the recipients of incremental HD over 12 months when compared to conventional care [20]. The number of cardiovascular events and deaths were low; one MACE was recorded in the intervention group and one death in the control group. This is consistent with previous observations reporting that participants in clinical trials of dialysis treatments tend to have a lower mortality risk than the general dialysis population [21].

This study has several limitations. It is not powered to show significant differences in outcomes, and, therefore, the findings reported here remain exploratory and will need further confirmation. The two treatment arms (intervention and control group patients) did not receive dialysis treatment contemporaneously; although dialysis practices did not change at our centre in the period covering the course of the entire study, factors such as staffing and support services available for participants may have been different. We did not have data to compare differences in staffing or resources spent per participant between these periods.

Recruitment and follow-up of participants was severely impacted by the events of the COVID-19 pandemic, which affected staffing and resources available. Whilst many studies at our institution had to be stopped, the current study was allowed to continue for existing participants since it involved the administering of a life-sustaining treatment (dialysis). The study could only be carried out, however, with significant limitations designed to minimise contact with participants. Monitoring of treatments was done remotely through dialysis treatment databases; research visits to the dialysis unit were kept to a bare minimum and only to ensure safety when clinical input was necessary; strict dialysis unit protocols were followed to minimise contact.

Our approach of starting patients on dialysis in a phased manner reflects a highly pragmatic approach to dialysis prescription, focusing on reducing the burden of early treatment and establishing patients on long-term dialysis with stepped and pre-planned increments. By doing so, its aim is to enhance patient experience of dialysis care at the start and help reduce the risk of physiological (in particular, cardiovascular) decompensation—described by Torreggiani et al. [4] as “dialysis shock”—which may be behind the high early mortality rates.

## 5. Conclusions

In conclusion, the current feasibility was designed to prepare the short transitional regime of incremental HD presented here, and shows promise when compared to conventional treatment; there were no signal for harm and, indeed, positive signals for certain outcomes such as a slower rate of decline in blood pressure, fewer episodes of intra-dialytic hypotension, and better preservation of vascular access. The aim of the current study was to prepare the ground for a future full-scale RCT, and the secondary outcomes presented here should be considered exploratory. These findings therefore provide a strong foundation for future testing of the programme in a definitive RCT.

## Figures and Tables

**Table 1:** Adverse events (AE) and serious adverse events (SAE) definitions. CV, cardiovascular; MI, myocardial infarction; HD, haemodialysis/haemodiafiltration; K, potassium; BP, blood pressure; SBP, systolic blood pressure; DBP, diastolic blood pressure (reproduced from Hazara et al., 2021, [10]).

Code	Name
SAE 1	Death from any cause
SAE 2	Major adverse cardiovascular events (4p-MACE: CV death, nonfatal MI, nonfatal stroke, hospitalisation for unstable angina)
SAE 3	Hospitalisation
SAE 4	Prolongation of existing hospitalisation
SAE 5	Leads to permanent disability
AE 1	Infections (if leads to hospitalisation)
AE 2	Infections treated as outpatients: any antibiotic treatment in the first 6 months of starting HD
AE 3	Intra-dialytic hypotension: any drops in BP of >20 mmHg systolic associated with nursing interventions (e.g., stopping ultrafiltration, lying the patient supine, or fluid bolus)
AE 4	Access problem 1: complete loss of access which cannot be used for dialysis
AE 5	Access problem 2: fistula required intervention
AE 6	Access problem 3: fistula required resting (for any reason)
AE 7	Hyperkalaemia 1: Any pre-HD ≥ 6.5
AE 8	Hyperkalaemia 2: two consecutive pre-HD K > 5.5
AE 9	Severe hypertension: pre-HD SBP > 180 or DBP > 110
AE 10	Interdialytic weight gain ≥ 4 KG
AE 11	Missed planned dialysis session for any reason
AE 12	Any other event which investigator believes may be linked to patient participation in the study

**Table 2:** Baseline characteristics of participants in the intervention and control groups. BP: blood pressure; eGFR: estimated glomerular filtration rate; SD: standard deviation.

	Intervention Group (*n* = 15)	Controls (*n* = 29)	Standard Mean Difference
Age, years, mean (SD)	62.8 (15.2)	58.9 (15.0)	0.25
Male, *n* (%)	10 (66.7%)	15 (51.7%)	0.31 *
Previous specialist input, months, mean (SD)	64.1 (53.4)	47.7 (40.1)	0.35 *
Permanent vascular access, %	93.3%	96.6%	0.14
Blood pressures (BP), mmHg			
Systolic BP, mean (SD)	158.4 (27.8)	157.7 (28.9)	0.03
Diastolic BP, mean (SD)	73.7 (8.9)	72.4 (15.7)	0.12
Body mass index, kg/m^2^, mean (SD)	32.2 (8.6)	34.4 (11.4)	−0.21
eGFR, ml/min/1.73 m^2^, mean (SD)	8.7 (3.1)	8.0 (1.3)	0.29
Haemoglobin, g/L, mean (SD)	97.4 (13.4)	99.2 (15.8)	−0.13
Albumin, g/L, mean (SD)	33.3 (4.8)	33.3 (5.2)	0.01
Parathyroid hormone, pmol/L	37.0 (27.8)	38.5 (23.2)	−0.06
Adjusted calcium, mmol/L, mean (SD)	2.3 (0.1)	2.3 (0.2)	−0.13
Phosphate, mmol/L, mean (SD)	1.6 (0.4)	1.5 (0.4)	0.41
Modality, %			0.07
Haemodiafiltration, %	80.0%	82.8%	
Haemodialysis, %	20.0%	16.7%	

* Gender and duration of previous specialist input were not part of matching criteria (see Methods section). Standard mean differences for these variables are presented here for consistency.

**Table 3:** Adverse events, hospitalisations, and deaths in participants of intervention and control groups. Outcomes are reported as total ‘number of events’ in each group, the ‘number of unique participants’ affected by these events (some participants experienced more than one event), and incidence rate of these events per group expressed in 100 person-year.

	Intervention Group (*n* = 15)	Controls (*n* = 29)
**Follow-up, person-year**	7.1	13.7
Infections treated as outpatient *		
Number of events	4	14
Number of unique participants	3	9
Incidence, 100 person-year	56.3	102.4
**Intra-dialytic hypotension**		
Number of events	15	48
Number of unique participants	7	18
Incidence, 100 person-year	211.0	351.1
**Complete loss of access**		
Number of events	4	11
Number of unique participants	3	9
Incidence, 100 person-year	56.3	80.5
**Fistula require resting**		
Number of events	4	4
Number of unique participants	4	4
Incidence, 100 person-year	56.3	29.3
**Hyperkalaemia 1 (Any potassium > 6.5)**		
Number of events	0	0
Number of unique participants	0	0
Incidence, 100 person-year	0	0
**Hyperkalaemia 2 (two consecutive potassium > 5.5)**		
Number of events	1	2
Number of unique participants	0	2
Incidence, 100 person-year	14.1	14.6
**Severe hypertension (SBP > 180 or DBP > 110) at any session**		
Number of sessions sampled	992	2044
Number of events	103	168
Number of unique participants	12	20
Incidence, 100 person-year	1448.6	1229.0
**Missed sessions for any reason**		
Number of events	7	12
Number of unique participants	4	7
Incidence, 100 person-year	98.5	87.8
**Hospitalisations**		
Number of events	9	19
Number of unique participants	5	13
Incidence, 100 person-year	126.6	139.0
**NSTEMI—survived**		
Number of events	1	0
Percentage of participants affected	7%	0%
Incidence, 100 person-year	14.1	0
**Deaths**		
Number of events	0	1
Percentage of participants affected	0%	3.4%

* Infections requiring hospitalisation were recorded separately (see hospitalisations).

## Data Availability

The original contributions presented in this study are included in the article/Supplementary Materials. Further inquiries can be directed to the corresponding author.

## Supplementary Materials

The following supporting information can be downloaded at: https://www.mdpi.com/article/10.3390/healthcare14091117/s1, Figure S1: Timeline for enrolment of participants in the intervention arm; Table S1: Eligibility criteria for study participants; Table S2: the TIDieR checklist; Table S3: Adverse events definitions; Table S4: Serious adverse events; Table S5: Reasons for hospitalisations.
