# Supplementary material for: Indicators of Safety and Wellbeing in Patients Starting Maintenance Haemodialysis Using Phased Approach: Findings from a Cohort Feasibility Study
Source: Healthcare (Basel). 2026 Apr 22;14(9):1117. doi: 10.3390/healthcare14091117 (PMC13163635; doi:10.3390/healthcare14091117)
Supplement: Supplementary file 1 [file healthcare-14-01117-s001.zip › supplementary tables.pdf]

Supplementary Table S1. Eligibility criteria for study participants.

Inclusion criteria	Exclusion criteria
1. Age ≥ 18<br>2. patients with CKD-5 who are about to start planned HD<br>3. At least 3 months of prior specialist renal follow-up at the time of starting HD<br>4. Able to meet all the study requirements<br>5. Written signed informed consent.	1. Age < 18<br>2. No prior contact with nephrologists for > 3 months<br>3. Cross-over into HD from peritoneal dialysis<br>4. Currently undergoing HD therapy<br>5. Any condition which in the opinion of the investigator makes the participant unsuitable for entry into the study<br>6. Participation in an interventional study in the preceding 6 weeks<br>7. History of myocardial infarction in the preceding 3 months<br>8. Inability to provide informed consent<br>9. Inability to comply with the study schedule and follow-up.

CKD: chronic kidney disease; HD: haemodialysis.

Supplementary Table S2. the TIDieR checklist

**The TIDieR checklist (the headings in bold represent items in the checklist)**
1. **Brief name:** Transitional incremental haemodialysis<br>2. **Why:** A pragmatic method of reducing exposure to dialysis during the early days of treatment in patients with established kidney failure, and to reduce early patient exposure to the risks posed by more intense dialysis at a time when patients are still adapting to the demands of regular dialysis. In these early days, less frequent dialysis may be sustainable as patients still retain residual kidney function.<br>3. **What – Materials:** Dialysis machine able to deliver haemodialysis or hemodiafiltration, high-flux synthetic dialyser, ultra-pure water and

replacement fluid produced on-line, bicarbonate buffer, arteriovenous fistula needle 16 gauge or above, patient vascular access must be able to provide at least 250 ml/min blood flow with <15% recirculation. Minimum 90 days of pre-dialysis specialist care a pre-requisite for participation to ensure dialysis preparation.

4. **What – Procedures:**
    a. Pre-dialysis: at each dialysis session, record pre-dialysis weight, blood pressure whilst patient is seated and enquire about general wellbeing (as in routine practice). Pre-dialysis serum urea and potassium checks at 1st, 4th, 5th, 6th sessions, then at 1- months since the start of dialysis, then monthly thereafter (laboratory request forms pre-provided). Measure overhydration using bioimpedance before session 1st, 4th, 6th sessions, then at 1- months since the start of dialysis, then monthly thereafter. Adjust target dry weight appropriately (or discuss at next multidisciplinary team meeting if uncertain). Instructions on taking bioimpedance readings presented separately.
    b. The dialysis treatment: use HD or HDF mode. Use 16G needle or larger gauge if using arteriovenous fistula. Commence dialysis in the usual way. Set up pump speeds as per usual practice. If fluid removal requirements exceed 1000 ml/hour, extend dialysis time to achieve specified target weight. Aim to achieve blood flows of > 250 ml/min.
    c. Post-dialysis: record weight and blood pressure, enquire about general wellbeing (as in routine practice). Post-dialysis bloods 'urea and electrolytes' at 1st session and monthly thereafter. Following the end of 1st dialysis session, send patient home with 24-hour urine collection bottle with standard instruction (available with the bottles). On return, weight sample, then send to laboratory and request urine volume, urine urea and urine creatinine.
    d. Ask for medical review if at any dialysis session a) an elevated potassium of > 6.5 mmol/l is detected, b) pre-dialysis systolic blood pressure is > 180 mmHg or diastolic blood pressure is > 110 mmHg, c) patient has gained 4 kg in weight since the end of previous dialysis session (inter-dialytic weight gain) or, d) if concerned about patient wellbeing in anyway.
5. **Who provided:**
    a. Ask for medical review if at any dialysis session a) an elevated potassium of > 6.5 mmol/l is detected, b) pre-dialysis systolic blood pressure is > 180 mmHg or diastolic blood pressure is > 110 mmHg, c) patient has gained 4 kg in weight since the end of previous dialysis session (inter-dialytic weight gain) or, d) if concerned about patient wellbeing in anyway.

   b. Dialysis specialist registered nurse able to provide haemodialysis treatments and experienced in dealing with immediate complications.
   c. Doctor (grade: specialist trainee year 3 or above) to review patient at 1st, 4th and 6th dialysis sessions, then at 1- months after the start of treatment then at monthly intervals. The doctor will use results of potassium checks, measurements of fluid load, residual urine output and clinical assessment to decide, in consultation with supervising consultant if appropriate, the course of ongoing treatments. Medical reviewer may decide keep patients on the incremental regime, prolong a given session or add one-off session(s) to correct the course of treatment, or to take patient off the incremental regime altogether.
   d. Multidisciplinary team (MDT) meeting: all patient evaluations/results to be discussed at the monthly dialysis MDT meeting in the presence of the supervising consultant.
6. **How:** Patients to attend dialysis centre to receive treatments, either Monday/Friday or Tuesday/Saturday if on twice-weekly regime or Monday/Wednesday/Friday or Tuesday/Thursday/Saturday if on three-times weekly treatments. Patient transport needs to be notified of the dialysis plan in advance (for patients requiring hospital transport to attend dialysis). Patients are given a copy of their dialysis schedule in the form of a calendar.
7. **Where:** Dialysis centre (main or satellite), equipped as per section 3 (see above), having facilities and skills to take blood samples from dialysis lines, able to periodically measure 24-hour urine outputs and perform bio-impedance tests.
8. **When and how much**
   a. The timing for starting dialysis: same as in routine practice, following clinical practice guidelines.
   b. Start twice weekly for two hour per session for 2 weeks, then three hours twice weekly for six weeks and then three hours three-times weekly for a further six weeks, then patients to change to a maintenance regime including three-times weekly four-hour sessions or residual function adjusted twice weekly regimes (see the figure in the main text).
9. **Tailoring:** Changes to the regime described in 8b may be made at any time to suit patient requirements as recommended at MDT meetings or following medical reviews. Such changes may also be made in emergencies (e.g., symptomatic fluid overload, hyperkalaemia > 6.5 mmol/l, or fistula abruptly fails). These changes may be in the form of extension to the dialysis time, recalling patients for additional dialysis session as a one-off,

escalating treatment to longer therapy times ahead of schedule or changing treatment times/frequency to conventional therapy.

Supplementary Table S3. Adverse events definitions

Code	Name	Method of data collection in treatment group	Method of data collection in treatment group
AE1	Infections (if leads to hospitalisation)	Record as SAE3 (see above)<br>Complete access database record	Complete access database record
AE2	Infections treated as outpatients	Search Lorenzo, SCR and Euclid for recorded treatment (any antibiotic treatment in the first 6 months of starting HD)<br>Enter in to access database	As with treatment group.<br><br>Enter in to access database
AE3	Intra-dialytic hypotension	Obtain records of all BP measurements from Euclid/therapy monitor. Identify all drops in BP of > 20 mmHg systolic. Then associate these with nursing interventions (e.g. stopping UF or fluid bolus)	Obtain records of all BP measurements from Euclid/therapy monitor. Identify all drops in BP of > 20 mmHg systolic. Then associate these with nursing interventions (e.g. stopping UF or fluid bolus)
AE4	Access problem 1: complete loss of access	From Euclid, see documentation of change from fistula to CVC and then confirm BHLY/Lorenzo	As with treatment group
AE5	Access problem 2: fistula required intervention	Radiology records, document reason for intervention	Radiology records, document reasons for intervention

AE6	Access problem 3: fistula required resting (for any reason)	Euclid and BHLY documentation (look out for unexplained gaps in dialysis)	Euclid and BHLY documentation (look for unexplained gaps in dialysis)
AE7	Hyperkalaemia 1	Any pre-HD ≥ 6.5	Any pre-HD ≥ 6.5
AE8	Hyperkalaemia 2	Two consecutive pre HD K > 5.5	Two consecutive pre HD K > 5.5
AE9	Severe HTN	Pre HD BP > 180 or DBP > 110	Pre HD BP ≥ 180 or DBP ≥ 110
AE10	Fluid overload	Inter-dialytic weight gain ≥ 4 KG	Inter-dialytic weight gain ≥ 4 KG
AE11	Missed planned dialysis session, any reason	From Euclid records	From Euclid records
AE12	Other event which investigator believes may be linked to patient participation in the study	Only relevant in treatment group	Not relevant (will not be compared)

Supplementary Table S4. Serious adverse events

Code	Name	Method of data collection in treatment group	Method of data collection in treatment group
SAE1	Death from any cause	Lorenzo	Lorenzo
SAE2	Major Adverse cardiovascular events (4p-MACE: CV death, nonfatal MI, nonfatal stroke, hospitalization for unstable angina)	Lorenzo. Paper case notes if needed.	Lorenzo. Paper case notes if needed.
SAE3	Hospitalisation	Lorenzo. Paper case notes if needed.	Lorenzo. Paper case notes if needed.

SAE4	Prolongation of existing hospitalisation	Lorenzo. Paper case notes if needed.	Lorenzo. Paper case notes if needed.
SAE5	Leads to permanent disability	Lorenzo. Paper case notes if needed.	Lorenzo. Paper case notes if needed.

Lorenzo: patient's electronic health record system

Supplementary Table S5. Reasons for hospitalisations

	Incremental HD group (n=15)	Control (n=29)
**All breathlessness**	**1**	**2**
- Breathlessness - pleural effusion		*1*
- Breathlessness - pulmonary oedema		*1*
- Breathlessness - Pneumothorax	*1*	
**All Chest pain**	**2**	**0**
- Chest pain – musculoskeletal	*1*	
- Chest pain – non-ST elevation MI	*1*	
**Urgent sort out of dialysis access**	**0**	**5**
**All infections**	**5**	**9**
- Infection - bacteraemia		*1*
- Infection – pneumonia (non-COVID-19)	*1*	*1*
- Infection - COVID-19	*1*	
- Infection - dialysis line	*1*	*1*
- Infection – finger		*2*
- Infection - gastroenteritis	*1*	
- Infection - leg (peripheral vascular disease)		*1*
- Infection - coexisting PD tube		*1*
- Infection - source unknown	*1*	*2*
**Other**	**1**	**3**

- Mechanical fall		*1*
- Palpitations	*1*	
- Severe Hypertension		*1*
- Hypoglycaemia		*1*
