# Supplementary figures and images for: Indicators of Safety and Wellbeing in Patients Starting Maintenance Haemodialysis Using Phased Approach: Findings from a Cohort Feasibility Study
Source: Healthcare (Basel). 2026 Apr 22;14(9):1117. doi: 10.3390/healthcare14091117 (PMC13163635; doi:10.3390/healthcare14091117)

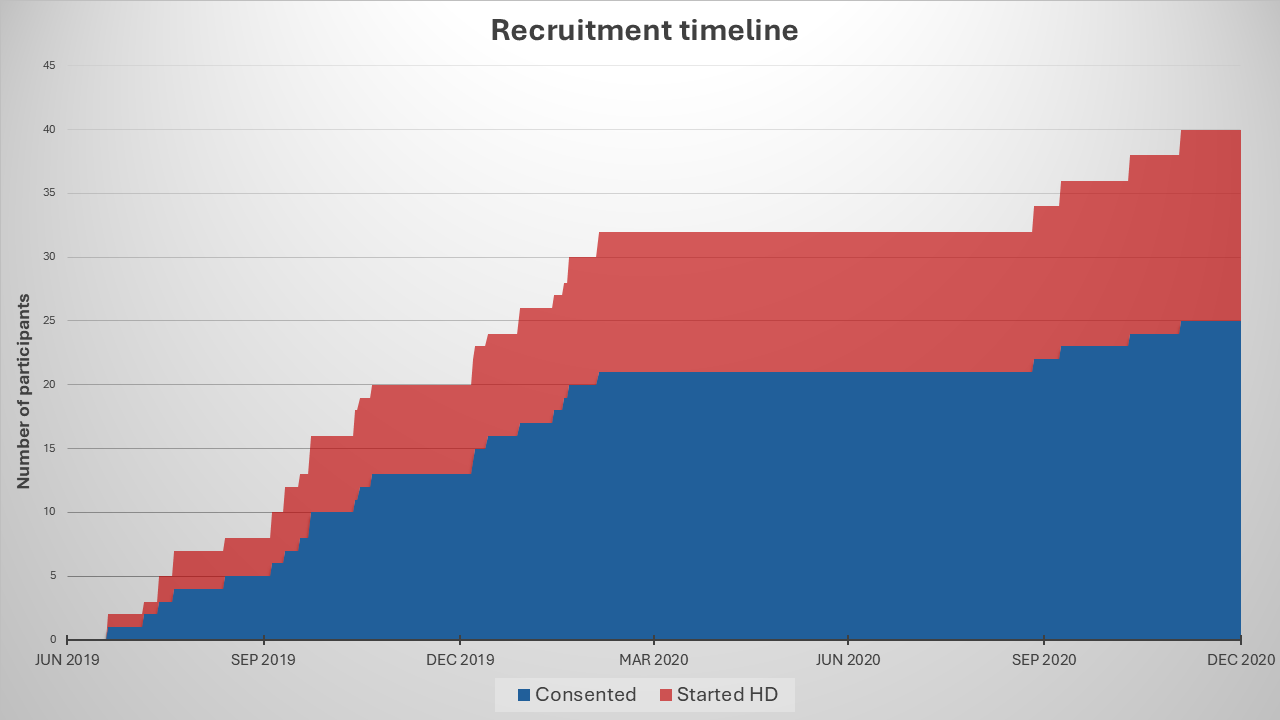

Supplement: Supplementary file 1 [file healthcare-14-01117-s001.zip › Figure S1.png]
